# Can We Identify Patients in Danger of Delayed Treatment? Management of COVID-19 Pandemic Backlog in Urology Care in Poland

**DOI:** 10.3390/ijerph192416547

**Published:** 2022-12-09

**Authors:** Jakub Marek Ratajczak, Anna Gawrońska, Margaret Fischer, Taras Hladun, Michał Marczak

**Affiliations:** 1 Department of Management and Logistics in Health Care, Medical University of Lodz, 90-647 Lodz, Poland; 2 Łukasiewicz Research Network, Poznań Institute of Technology, 61-755 Poznań, Poland; 3 Faculty of Pharmacy, Medical University of Gdansk, 80-210 Gdansk, Poland; 4 Urology Department, Regional Specialized Hospital in Nowa Sól, 67-100 Nowa Sól, Poland

**Keywords:** urology, oncology, COVID-19, backlog, urolithiasis

## Abstract

The COVID-19 pandemic had a tremendous impact on healthcare systems around the world. This study aims to research the course of surgical treatment in urology during the pandemic in 2020, evaluate the volume of deferred treatment in urology in Poland, and indicate groups of patients that are especially vulnerable to a delay in the delivery of healthcare services. The National Health Found statistics (NHF) database was searched for information on procedures completed in urology departments from 2015 to 2020. Changes in hospital discharges of adults from 2019 to 2021 were investigated using monthly reports of NHF on patient billing groups. Statistics of PSA, testosterone, and creatinine testing were extracted from NHF reports. Annual changes in the number of surgeries were calculated. Then, the estimation of the expected quantity of procedures without the occurrence of the pandemic was performed using linear regression based on data from 2015 to 2020. The estimation was assumed reliable at R^2^ > 0.8. The difference between collected and estimated data was analysed. In 2020, the volume of radical prostatectomies, cystectomies, and kidney surgeries noted downturns following lockdowns in March and November. All analysed procedures, except radical cystectomy, noted a reduction in the entire year. The declines reached −34% in shockwave lithotripsy, −13% in ureterorenoscopic lithotripsy, −22% in cystolithotripsy, −28% in percutaneous lithotripsy, −12% in transurethral resection of a bladder tumour (TURBT), −31% in transurethral resection of the prostate, −15% in nephrectomy and kidney tumorectomy, and −10% in radical prostatectomy. Among the analysed procedures, only radical cystectomy rates increased 5%. Prostate-specific antigen and creatinine tests fell −17%, and testosterone testing was down −18%. In conclusion, the patients most vulnerable to delayed treatment due to the post-pandemic backlog are those requiring TURBT, kidney cancer operations, and radical prostatectomies. Solving backlogs in urology should prioritise cancer patients and thus requires improved access to cystoscopy, TURBT, diagnoses and surgery of prostate and kidney tumours. Addressing the needs of patients suffering from benign diseases demands appropriate measures to increase the surgical productivity of urology departments.

## 1. Introduction

The pandemic has changed the functioning of medical care all over the world. The very first reports of pneumonia of unknown origin began to reach Poland at the end of 2019. On 9 January 2020, the World Health Organization (WHO) announced that Wuhan, China, was suffering from mysterious pneumonia related to an infection with an unknown type of virus, and on 12 January 2020, the genetic sequence of the new coronavirus was decoded and made available to the rest of the world [1]. Reports of the first case of the new disease, known as COVID-19, were confirmed in Poland on 4 March 2020. On 10 March 2020, the first restrictions were introduced in Poland, including the cancellation of mass events. A day later, the WHO announced the global COVID-19 pandemic. 

The first lockdown began on 12 March 2020, with the closing of all schools in Poland. From there on, classes in schools and universities were held remotely. These restrictions also impacted various activities of public and cultural institutions, which followed their temporary suspension, as the regulation of the Minister of Health confirmed the spread of the epidemic in Poland.

On 16 April 2020, nose and mouth coverings were compulsory in public places. About a month later, when the situation was recognised, the gradual lifting of restrictions began across Poland and continued until 9 November 2020, when schools were closed again, bringing about the beginning of the second lockdown. Daily death tolls were soaring, with a record high noted on 25 November 2020 at 674. According to the Minister of Health, the epidemic state in Poland spanned from 20 March 2020 and ended after more than two years, on 15 May 2022. From 16 May 2022, the state of an epidemic threat was put in force. By 5 November 2022, there were 6,343,578 cases of infection, while 118,170 deaths were registered [2]. In the first year of the pandemic, there was a 16% increase in total mortality, and in the population, those over 65 years of age were found to be most vulnerable [3]. The estimated number of excess deaths due to COVID-19 has varied from 68,505 to 214,000 [4,5].

In 2019, the waiting lists for hospital admission in urology departments were 23.2% higher than the year before, which means that the public health service was already strained before the pandemic began [6]. The flow of patients in the Polish healthcare system is divided into three streams: urgent, oncology, and stable (elective). The outbreak of COVID-19 froze the admissions of the first group, leaving space for oncology and urgent groups. The decision was upheld during the following lockdowns. Observing their toll on elective urology care allowed us to assume that systemic problems in hospital treatment will accumulate. Although priority was given to cancer patients, it was not clear if reduced access due to COVID-19 will be sufficient for the demand. Clinicians suspect the gap is widening, with a growing number of respiratory tract urgencies. According to the WHO International Agency for Research and Cancer, in 2018, the risk of developing cancer in the Polish population prior to age 75 was 30.0% in men and 23.3% in women, whereas the risk of dying from malignancy under the same age was 18.9% and 11.5%, respectively. The highest morbidity in females was caused by non-urological tumours: breast, lung, colorectum, corpus uteri, and ovarian cancer. The most prevalent cancers in men include the lung, prostate, colorectum, bladder, and stomach. Prostate and bladder cancers are the fourth and fifth most frequent in the general population. The number of all oncology cases in Poland in 2019 reached 171,218 [7]. In males, new cases of urological neoplasms reported in 2018 were as follows: prostate, 15,393; bladder, 10,883; kidneys, 6327; testis, 1417; penis, 395. Age-adjusted incidence rates for men per 100,000 inhabitants (European Standard Population, revised 2013) in 2017 were 114.8 for prostate cancer, 39.9 for bladder cancer, and 20.2 for kidney cancer [8]. The last two decades have shown a rapid increase in the morbidity of prostate cancer, while incidences of kidney and bladder cancer have remained stable. COVID-19 has had an unprecedented influence on urology in Poland; however, changes during the pandemic and its possible long-term effects have not yet been sufficiently studied.

The aim of this study is to research the course of surgical treatment in urology during the pandemic in 2020. The second goal is to analyse the size of the pandemic backlog in urology surgical treatment. The third is to identify patients who may be at risk of delayed care and indicate necessary steps to minimise a possible burden of diseases.

## 2. Materials and Methods

The National Health Fund statistics (NHF), an online database of the public healthcare provider in Poland, was used to obtain information on inpatient urological procedures completed from 2015 to 2020. Monthly reports of NHF on patient billing groups were processed to collect data on hospital discharges from 2019 to 2021. Information was retrieved by procedure codes published in the Polish version of International Classification of Diseases, Ninth Revision (ICD-9 PL), disorders’ names listed in International Statistical Classification of Diseases and Related Health Problems (ICD-10) and billing groups. Procedure names and codes are presented in Table 1. Investigated population covered only adult patients. Statistics of PSA, testosterone and creatinine testing were extracted from specific NHF reports. Year-to-year change in number of procedures was calculated. Projection on volume of services without occurrence of COVID-19 epidemic was performed, and then the difference between estimation and factual data was analysed. Influence of the pandemic on the volume was modelled using linear regression based on data from 2015 to 2020. Reliable estimation was assumed at R^2^ > 0.8. Computation was made with Microsoft Excel 2021 (Redmond, WA, USA).

## 3. Results

### 3.1. Urological Cancer Surgery in the Pandemic

#### 3.1.1. Radical Prostatectomy

**Figure 1:**
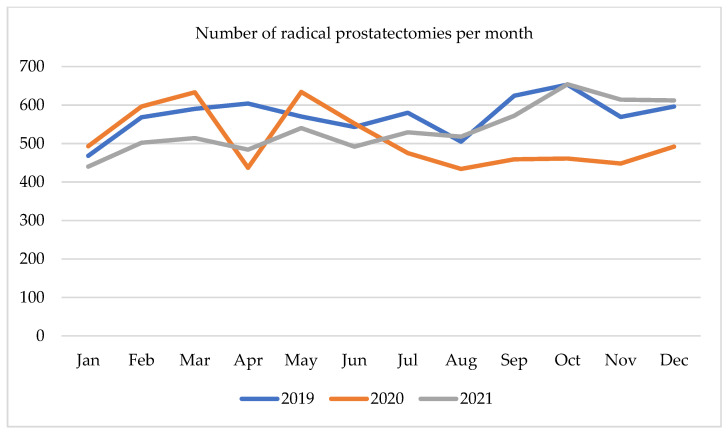
Number of radical prostatectomies per month in 2019–2021.

The first three months of 2020 showed a steady increase in the number of radical prostatectomies (RP), with a sharp decline of 30% from 633 to 437 operations recorded in April 2020. In the next month, the total number of surgeries rapidly returned to almost the same level as previously (634). Over the following three months, the volume of prostatectomies was gradually falling, reaching a plateau of around 500, which fluctuated until Autumn 2021. In October 2021, it again reached a maximum of 654 and remained over 600 until the end of the year. Figure 1 highlights the monthly number of radical prostatectomies.

#### 3.1.2. Radical Cystectomy

**Figure 2:**
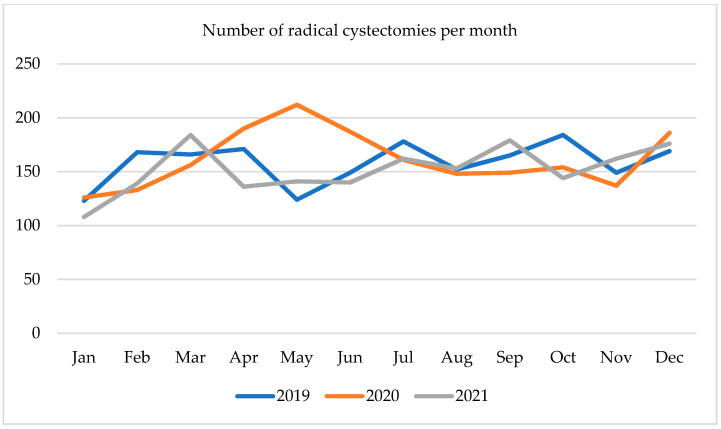
Number of radical cystectomies per month in 2019–2021.

Since the beginning of 2020, the number of radical cystectomies (RC) was increasing from 126 in January 2020 to 212 in May 2020. Over the next 3 months, it dropped to 148 and remained at that level until November 2020, when the number of cystectomies reached the level of 137. Although in December 2020, the number of surgeries raised to 186, it then fell to 108 in January 2021. In the first 3 months of 2021 number of RC reached 184, then it declined to 136 and rose again towards the end of the year with a spike in September 2021 to 179. The monthly number of radical cystectomies is presented in Figure 2.

#### 3.1.3. Kidney Surgery

**Figure 3:**
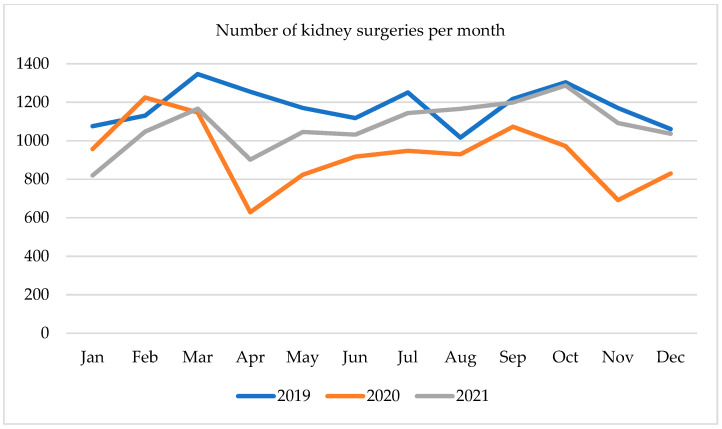
Number of kidney surgeries per month in 2019–2021.

The volume of hospital discharges due to kidney surgery in the first months of 2020 was seasonally fluctuating; however, a major downturn was recorded in April 2020 (Figure 3). Annual fall was at 49%, whereas a month-to-month decrease was at 45%, amounting to 629 surgeries. In the following months, an upturn trend reached a plateau at a level of 918–948 operations in the summer of 2020. Then a second drop was observed in November 2020, falling by 40% annually to 692 operations. At the end of the year number of surgeries increased until March 2021, when it reached 1167 discharges. In April 2021, a moderate decline to 902 was registered, which was more than the previous year, and yet 28% less than in 2019. Later in 2021 number of kidney operations was steadily increasing, but the trend line remained lower than in 2019.

### 3.2. Urological Procedures in Past 5 Years and Projection for 2020

#### 3.2.1. Urolithiasis Treatment

Shockwave lithotripsy (SWL) has been steadily falling since 2015, at an annual rate ranging from 6% to 16%. A major decrease of 34% was noted in 2020, where the expected value was 18%. This difference equals 2434 avoided sessions. Ureterorenoscopic lithotripsy (URSL) has been on a continuous rise in the past 5 years. In 2020 a significant downturn of 13% was noted, whereas disparity in the expected volume of surgeries reached 20%, which represents 4089 untreated patients. During the analysed period, a substantial shift in the proportion of operative methods occurred. Initially, the involvement of lithoclast dominated stone treatment, yet in 2018 it was superseded by laser lithotripsy, which almost doubled from 5884 to 10,222 in the last 5 years. Analysed records have shown a dynamic increase in retrograde intrarenal surgery (RIRS) from 397 to 3109 operations annually, which amounts to a 782% surge over 6 years. It was projected to rise by 21% in 2020, reaching 3765 surgeries, but eventually, the total volume of RIRS remained at the same level. A gradual drop in laparoscopic ureterolithotomy was observed, although a 48% decrease was far more profound than expected (−32%). The number of percutaneous lithotomies was slightly fluctuating between values of 3746 to 4009; however, in the pandemic year, there was a sudden decline to 2880, which is 1023 less than the anticipated 3903. The annual fall of 28% was much deeper than the previous variations (−5% to +7%). Bladder stone removal rate balanced at a level of 2834 to 3131, with a steep decline to 2420 in the year 2020. The gap of 682 cystolithotripsies means a 22% annual fall. Urological procedures in stone disease are displayed in Table 2.

#### 3.2.2. Functional Urology Treatment

The amount of TURP was steady in the stated period, with minimal changes of 1–2% (Table 3). Though, in 2020 there was a massive downturn of 31% from 13,550 to 9310, resulting in a gap of 4240 resections. UPJ reconstruction volume was gradually decreasing at a rate of 2% to 6% until 2018, when the trend accelerated to −33% in 2019 and continued in 2020 at a level of −23%.

#### 3.2.3. Cancer Treatment

The burden of transurethral bladder tumour resections (TUR-BT) was steadily rising 1 to 5% a year since 2015, reaching a volume of 37,123 surgeries in 2019. The following year brought a substantial decline of 12%, meaning 4611 missed resections. The predicted number of bladder tumour operations for 2020 was 38,115; therefore, the estimated volume of unperformed procedures equals 5603, which is 15% less than factual data from 2020. Radical cystectomy volume was alternating over the described period. In men, after the initial decrease in 2016–2019, an upturn of 7% occurred in 2020, which was the highest number in the presented timeframe, resulting in 1532 surgeries. In women, cystectomy rates varied from 393 to 466, while the peak was reached in 2019 and remained almost unchanged the following year, with 464 cystectomies performed. In total, the number of radical cystectomies differed from 1812 to 1996 a year, with a growth of 5% in 2020, which was more than the expected 1937 surgeries. 

The volume of kidney cancer operations has been steadily growing since 2015 at a pace of 1–5%, reaching 8051 in 2019. An unprecedented decline of 15% occurred in 2020. The number of nephrectomy and nephron-sparing surgeries (NSS) fell from 8051 to 6814, creating a gap of 1237 operations. 

Prostatectomy figures were rapidly moving up from 4677 to 6879 until 2019. The following year brought a fall of 10% to 6185. However, a projection revealed a deeper decrease of 20%, as it was expected to have performed 7718 surgeries. This means the difference between the number of completed operations and estimation is 1533. Table 4. contains detailed data on cancer treatment.

##### PSA, Testosterone, and Creatinine Testing 2016–2021

**Figure 4:**
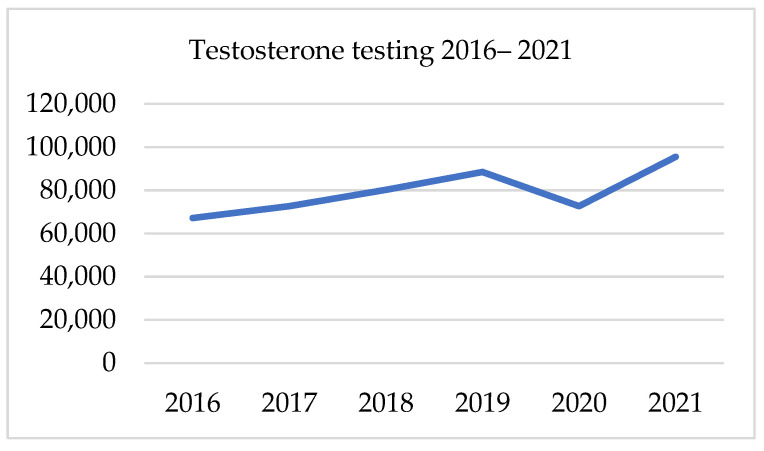
Testosterone testing in 2016–2021.

**Figure 5:**
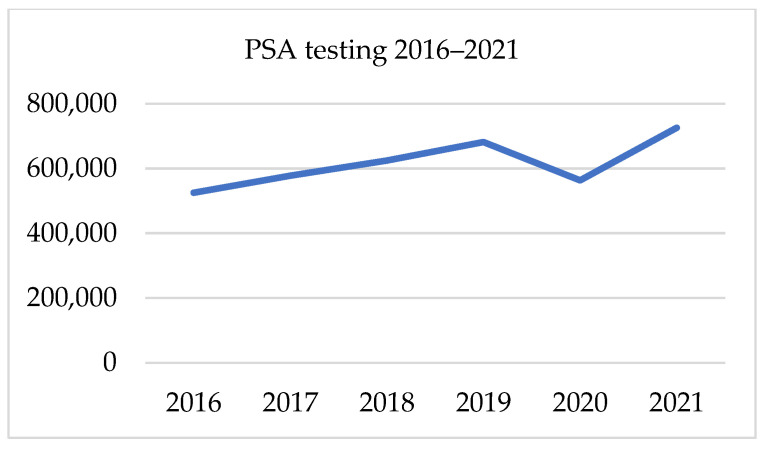
PSA testing in 2016–2021.

**Figure 6:**
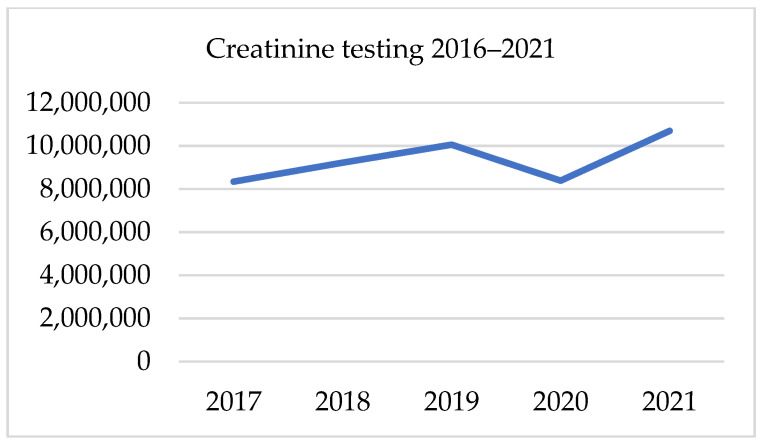
Creatinine testing in 2016–2021.

There has been a gradual growth in the testing of PSA, creatinine and testosterone since 2016. Those three parameters followed the same trend in the presented period. From 2017 to 2019, the average annual growth rate was 9.1% for PSA, 9.6% for testosterone and 10.6% for creatinine. In 2020 there was a significant decline in all tests, which was proportional in all three factors at an annual drop of 17–18%. In 2020 the gap in the number of PSA was 118 187 samples. The following year total number of PSA tests was 29% higher, though it was only 6% higher than in 2019. Likewise, the volume of testosterone sampling in 2021 was 7.9% higher than in 2019. Regarding the number of creatinine tests, in 2021, there was a 28% increase after its fall of 17% in 2020. Nevertheless, this result was only 6.4% higher than 2 years earlier. Table 5 and Figure 4, Figure 5 and Figure 6. presents data on laboratory testing.

##### Kidney Cancer—Summary of Hospitalisations

The volume of hospitalisations due to renal cell and urothelial cell kidney cancer was progressing over the analysed period. Projection revealed a large downturn of 8.18% in 2020, meaning approximately 3584 fewer discharges. The deepest fall was observed in the surgical treatment; the number of not performed operations is estimated to be as high as 927. The second most affected area was chemotherapy, where 8.56% fewer patients were admitted. Other treatment modalities, including radiotherapy, immunotherapy and palliative care, also noted declines of 4%, 5% and 2.5%, respectively. Detailed data are presented in Table 6.

##### Bladder Cancer—Summary of Hospitalisations

The number of hospital admissions due to bladder cancer was steadily growing in the presented period (Table 7). In 2020 there was a downturn in all areas of treatment. In total, the number of discharges was estimated to fall by 7.81% to expected values for 2020, with the deepest drop in chemotherapy (−12.73%) and surgical care (−12.26%). Radiotherapy hospitalisations and the number of patients receiving chemotherapy decreased by 10.42% and 7.38%, respectively.

## 4. Discussion

In response to the pandemic, numerous Polish hospitals and their urology departments were converted into dedicated centres for COVID-19. Despite the strain put on the hospitals, that course of action was necessary at the time, which further brought about a reduction of specialist wards and impacted urology treatment in hospitals.

This was also echoed in worldwide surveys carried out in the spring of 2020, which reported that 26% of respondents claimed that urologists were deployed to treat COVID-19, and 67% declared that elective procedures were stopped or reduced by over 75% [9,10]. During the whole period of the pandemic, elective operations were postponed on a regular basis. The oncology treatments were given priority, while the healthcare system capacity was occupied with the COVID-19 patients [11]. The everyday practice has been tremendously changed both in hospital and ambulatory care. The measures taken to advance patient safety triggered a prompt application of telemedicine and information technology systems, which otherwise would have been pending their implementation for another decade [12,13,14,15,16].

All non-urgent urolithiasis procedures were suspended during the lockdowns, which in turn had a significantly negative impact on the admission statistics in 2020. In general, the volume of all treatment methods has decreased. The SWL rates dropped by 34%, which was more than expected. As a result, almost 5300 sessions were missed. As a long haul consequence, this may lead to some patients potentially having invasive procedures such as URSL in the future due to stones becoming symptomatic or too large for shock wave treatment. As a repercussion of these actions, this may cause an additional strain on already long queues for an endoscopy. While there was a 7% increase in URSL expected, it was met with a 13% decline, which accounted for a gap of over 2000 operations. During the COVID-19 outbreak, many patients were unwilling to seek medical help, even in urgent situations. Thus, a drop in emergency department visits due to urological conditions was observed in the United States by 20%, 42% in Germany and 22% in Poland [17,18,19]. This may then explain the already noted decrease in surgeries for ureteral stones. Nevertheless, the long-term effect of untreated hydronephrosis or ureteral strictures might trigger a wave of patients requiring secondary procedures such as endoscopic incisions, reconstructions, or even nephrectomy. Eventually, some could also develop kidney insufficiency as a result. Over the presented period, the use of laser in ureteroscopy was so prevalent that it had superseded the harness of pneumatic lithoclast and laparoscopic ureterolithotomy. Another treatment method that was also on the rise and became widely available was the flexible ureteroscopy, which replaced the multiple SWL sessions and enabled an effective treatment [20]. While the RIRS is usually an elective operation, the rates remained similar in 2019 and 2020. This then contradicted the assumption that was based on the dynamic growth of the RIRS operations in the previous years, which was estimated to have increased by 21%. It is important to note that the pandemic also caused a steep reduction in the number of PCNL (−28%), leading to 1110 fewer procedures performed annually. The PCNL is a treatment method dedicated to sizeable or staghorn stones. Thus, longer waiting times may result in the formation of even larger stones, higher occurrence of sepsis in patients with infection stones, as well as subsequent nephrectomy due to the insufficiency, should the kidney finally deteriorate [21]. 

Major declines in the treatment of benign prostatic hyperplasia and ureteropelvic junction reconstruction reached 31% and 23%, respectively. During the pandemic lockdowns, these procedures were almost completely seized. There is no doubt that these conditions progress slowly, having no direct threat to the general health of individuals; however, the upsetting symptoms may appear to worsen patients’ quality of life [22,23]. Given the need to mitigate the backlog of oncology treatment, thus, access to urinary stones and functional urology services is still expected to remain far below demand. 

Trends of radical prostatectomy during the pandemic came after national lockdowns in Poland. A major fall in the number of operations was observed in April 2020, after the first lockdown in March 2020. The monthly admissions remained lower throughout the year and increased at the end of 2021, resulting in the reduction of prostate cancer operations, reaching 10% in the whole of 2020, which is much deeper than the 3.6% fall reported in France [24]. Having considered the increase in number of the prostatectomies in the previous years, it was estimated that 1533 were not completed due to the pandemic restrictions facing a shortage of intensive care beds and anaesthetists. Considering the biology and slow progression of prostate cancer, the European and Polish Urological Associations recommended postponing all prostatectomies in low-risk cancer patients over patients with more aggressive tumours [25,26]. These were also caused by the delay of the prostate biopsies, alongside the decreased PSA testing observed in 2020, which may have resulted in fewer referrals for the prostate biopsies and subsequently withheld cancer detection and treatment. Regarding the pandemic backlog in diagnostics and demographic factors, we may expect increased morbidity from prostate cancer; therefore, elevated demand for prostatectomy and pathology results in a higher grade of disease [27]. On the contrary, a 3 to 6 months delay of the radical prostatectomy was found to have had no negative impact on the recurrence rates; however, high-risk patients should not wait longer than the above-stated timeframe [28]. This could be diminished by more patients deciding on the active surveillance of the tumour and a higher number of those unfit for radical treatment due to the comorbidities and disease stage. The prostate MRI facilitates the targeted biopsy and enhances its accuracy, therefore reducing the requirement for multiple procedures and shortening the time for a proper diagnosis, as well as improving surgical planning [29,30,31]. In fact, a downturn in testosterone and the PSA testing meant that some patients had not received a prompt follow-up of prostate cancer. This may result in a later diagnosis of the relapse or metastases, which could otherwise be detected early by the wide use of positron-emission tomography with the prostate-specific membrane antigen (PET-PSMA) [32]. A creatinine test is another diagnostic parameter which has also seen its decline throughout 2020, even though it is commonly employed in various specialities of medicine. This downturn may have delayed the detection of chronic kidney disease, which could reveal itself in the coming years.

Patients suffering from bladder cancer (BCa) represent the largest group affected by the pandemic in urology. The study revealed an estimated decline of 12% in surgical hospitalisations and of 15% in the statistics of TURBT, interpreted for over 5600 endoscopic resections less than what would have been performed. A comparable drop in the number of TURBT, reaching 19% annually, was reported in Italian oncology centres [33]. On the other hand, in a country-wide study in France, the fall in endoscopic bladder tumour resection was only 2% [24]. Especially alarming is the fact that during the pandemic period, patients with primary tumours were found to be at a more advanced stage and higher grade of disease [34]. Therefore, a sound clinical judgement was necessary for those previously diagnosed with low-risk tumours and small recurrent lesions, where a deferred treatment was considered safe [35]. Given these factors, the number of bladder cancer patients with high-grade and muscle-invasive diseases may increase in years to come. Management of this problem will require improved access to diagnostic care, including cystoscopy, bladder biopsy and tumour resection. Furthermore, facing the Bacillus CalmettGuérin shortage may be aggravated by an increased demand [36]. Thus, prompt treatment in order to resolve the backlog is of utmost importance to diminish a possible escalation of healthcare expenses due to the following requirements of cystectomy, chemotherapy, and palliative care alongside its damaging social consequences. The radical cystectomy rates fluctuated in the presented period. The pandemic year showed a slight rise of 5% in total, in comparison to a 6% drop in the French study by Robert et al. [24]. The male population statistic rose by 7%, while in women, it remained unchanged. The strengthening effect of COVID-19 on cystectomy statistics might be down by the shift to oncological surgery and rescheduling of operations for benign diseases. It may only be assumed that the stream of patients with clear suspicion of potentially advanced disease was accelerated by the shorter waiting times for cancer diagnosis at the beginning of the pandemic. In addition, some patients and clinicians may have chosen an early radical treatment in order to avoid the uncertainty around healthcare access, admission rescheduling and subsequent poorer outcome of the treatment. All are weighted against multiple potential procedures required in high-grade non-muscle invasive bladder cancer and carcinoma in situ [37]. Moreover, the operations performed at the lower stage of the disease can reduce the occurrence of post-operative complications [38]. It is clear that the overall survival in BCa is worse if the waiting time for cystectomy is postponed over 3 months after TURBT [39]. It may be assumed that an upturn in the radical treatment observed in 2020 will improve the survival rates in the following years since many of these patients have undergone an early cystectomy and, this way, avoided a delayed operation [40,41]. However, the toll of missed transurethral resections might be seen at a later stage, resulting in more cystectomies and an upturn in mortality.

The annual number of operations and surgical hospitalisations due to kidney tumours, including renal cell carcinoma (RCC) and urothelial cancer, was continuously growing in the analysed period until a sudden downturn of 15% in 2020. Research in France revealed a 9% decrease in kidney cancer surgery [24]. The pandemic gap in the nephrectomy and NSS was estimated to reach 1237 cases. A typical course of RCC, characterised by slow growth and low potential of metastasis until large volumes of the tumour, allows for deferring the treatment or the use of ablation techniques [42]. Considering active surveillance can be an option for small renal cell tumours below 4 cm in diameter, which was also the prerequisite to applying the risk-adapted treatment strategy during the pandemic [43,44]. This explains the periodic fluctuations of kidney operations throughout 2020, which followed the national lockdowns in Poland. Although postponing the operations of small renal masses seemed justified at the time, it could have a negative impact on cancer-specific survival rates even in T1a (<4 cm) renal cell cancer [45]. In the upper tract urothelial tumours, waiting times exceeding 3 months are also associated with worse survival rates [46]. The repercussions of this might be seen in the coming years disguised as an increased number of patients receiving immunotherapy, palliative care and, finally, as a cause of death. Changes in mortality might be first seen in bladder cancer, as life expectancy in advanced disease is shorter than in kidney cancer [47].

Mitigation of the pandemic backlog requires an increased throughput of urology departments, which can be achieved by additional operation sessions and a higher volume of day-care surgery, especially TURBT for bladder cancer patients. In addition, better financing and purchase of novel, more effective equipment, e.g., high-power lasers and flexible ureteroscopes, would tackle the problem of multiple-stage procedures in the urinary stone treatment by increasing the volume of RIRS and holmium laser enucleation in benign prostate hypertrophy. Easy access to the targeted prostate biopsy may facilitate an early diagnosis of cancer, and further detection of relapse or metastases could be found faster by PET-PSMA. The introduction of the robotic sets may improve outcomes of radical prostatectomy and cystectomy. Finally, improved access to and efficiency of ambulatory care may reduce the need for hospital treatment.

## 5. Limitations of Study and Areas for Future Research

Regarding the limitations of this study, the latest cancer statistics for Poland were published in 2019, which was the sole reason for the investigation into The National Health Fund reports. Statistics of the procedures are updated annually; however, the currently available data only covers the year 2020, which is the most recent publicly available data on oncology treatment in Poland. On these grounds, the investigation was only possible for the first year of the pandemic. The monthly hospital discharge reports are presented in the billing groups, where kidney surgery is merged, mainly including cancer and urolithiasis treatment, without the ICD-9 procedures’ codes; however, a precise diagnosis along with the procedure codes is detailed in the annual summary. Data, however, does not include surgeries performed in the private sector.

The oncology statistics data published in the future will allow the verification of the efficiency of measures taken to save both oncology and COVID-19 patients. Changes in the patterns of cancer morbidity and mortality triggered by the pandemic can be investigated as well. This will also facilitate the adjustment of healthcare policy in emergency situations in the future. An application of new diagnostic methods and operative equipment should undergo the health technology assessment according to the standardised procedures.

## 6. Conclusions

On the one hand, the deferral of elective surgeries protected many patients from contracting the COVID-19 infection and directed more resources to treatment for the seriously ill. On the other hand, those who were advised to postpone the diagnostic measures or treatment may suffer from a more advanced form of the disease in the future. Consequently, more patients will suffer from more advanced cancers and require palliative care, which might be the hidden cost of the pandemic in both social and economic aspects. Unfortunately, patients referred due to non-malignant conditions may wait even longer for admission as elimination of the backlog in cancer treatment will be the priority. In many patients, the disease may progress and exacerbate symptoms, which will negatively impact their quality of life. The final effects of the pandemic will be seen over the next few years. Nevertheless, the appropriate measures should be taken expeditiously to minimise its adverse effects in the future.

## Figures and Tables

**Table 1:** Procedure names, codes and billing groups.

Procedure Name	Searched ICD-9/ICD-10 Codes/NHF Billing Groups
Shock wave lithotripsy (SWL)	98.51
Laser ureterorenoscopy (URSL)	56.023
Lithoclast URSL	56.022
Flexible URSL/Retrograde intrarenal surgery (RIRS)	56.024
Cystolithotripsy	57.031, 57.032, 57.033
Ureterolithotomy (laparoscopic)	56.021
Percutaneous nephrolithotomy (PCNL)	55.04, 155.042, 55.043, 55.03, 55.044
Transurethral resection of bladder tumour (TUR-BT)	57.421, 57.422, 57.49, 57.424, 57.423
Transurethral resection of prostate (TURP)	60.295, 60.231, 60.232, 60.212, 60.261, 60.291, 60.293
Nephrectomy and nephron sparing surgery (NSS)	L05, L00 for C64, D41, C65, C66
Ureteropelvic junction (UPJ) reconstruction	55.871, 55.872
Radical prostatectomy (RP)	L31
Cystectomy (RC)	L21, L22

**Table 2:** Urolithiasis procedures registered in 2015 to 2020 and expected values for 2020.

Procedure	2015	2016	2017	2018	2019	2020	Estimation for 2020	R^2^	Adjusted R^2^
SWL									
Total	24,335	21,907	19,583	16,505	15,499	10,209	12,643		
Annual change		−10%	−11%	−16%	−6%	−34%	−18%	0.9835	0.9794
URSL									
Laser	5884	7100	8210	10,413	10,916	10,222	12,517	0.9734	0.9667
Annual change		21%	16%	27%	5%	−6%	15%		
Lithoclast	10,059	10,556	10,050	9095	9647	7158	9195	0.4393	0.2992
Annual change		5%	−5%	−10%	6%	−26%	−5%		
RIRS	397	850	1321	2531	3099	3109	3765	0.9665	0.9581
Annual change		114%	55%	92%	22%	0%	21%		
Total	16,340	18,506	19,581	22,039	23,662	20,489	25,478	0.9902	0.9877
Annual change		13%	6%	13%	7%	−13%	8%		
Cystolithotripsy									
Total	2834	3087	3060	3131	3102	2420	3043	0.5888	0.4860
Annual change		9%	−1%	2%	−1%	−22%	−2%		
Laparoscopic ureterolithotomy									
Total	1179	908	712	696	605	312	412	0.8812	0.8515
Annual change		−23%	−22%	−2%	−13%	−48%	−32%		
PCNL									
Total	3819	4009	3950	3746	3990	2880	3903	0.0118	−0.2352
Annual change		5%	−1%	−5%	7%	−28%	−2%		

**Table 3:** Functional urology procedures registered in 2015 to 2020 and expected values for 2020.

Procedure	2015	2016	2017	2018	2019	2020	Estimation for 2020	R^2^	Adjusted R^2^
TURP									
Total	14,277	13,951	13,748	13,428	13,550	9310	13,197	0.8619	0.8273
Annual change		−2%	−1%	−2%	1%	−31%	−3%		
UPJ reconstruction									
Total	874	855	816	767	513	393	699	0.7621	0.7026
Annual change		−2%	−5%	−6%	−33%	−23%	36%		

**Table 4:** Urological oncology procedures performed in 2015 to 2020.

Procedure	2015	2016	2017	2018	2019	2020	Estimation for 2020	R^2^	Adjusted R^2^
TUR-BT									
Total	32,072	33,796	34,294	35,533	37,123	32,512	38,115	0.9760	0.9701
Annual change		5%	1%	4%	4%	−12%	3%		
Cystectomy									
Total (men)	1419	1515	1496	1461	1434	1532	1457	0.0088	−0.2390
Annual change (men)		7%	−1%	−2%	−2%	7%	2%		
Total (women)	393	413	451	431	466	464	480	0.7927	0.7409
Annual change (women)		5%	9%	−4%	8%	0%	3%		
Total	1812	1928	1947	1892	1900	1996	1937	0.1830	−0.0213
Annual change		6%	1%	−3%	0%	5%	2%		
Prostatectomy									
Men	4677	5685	6426	6739	6879	6185	7718	0.8981	0.8726
Annual change		22%	13%	5%	2%	−10%	12%		
Nephrectomy and NSS									
Total	7368	7483	7611	7989	8051	6814	7884	0.9421	0.9277
Annual change		2%	2%	5%	1%	−15%	−2%		

**Table 5:** Number of PSA, testosterone and creatine tests 2016–2021.

Year	PSA	Annual Difference	Annual Change	Testosterone	Annual Difference	Annual Change	Creatinine	Annual Difference	Annual Change
2016	525,130			67,157			741,8350		
2017	577,818	52,688	10%	72,684	5527	8%	834,018	922,668	12%
2018	624,493	46,675	8%	80,246	7562	10%	9,219,090	878,072	11%
2019	681,721	57,228	9%	88,500	8254	10%	10,050,681	831,591	9%
2020	563,534	−118,187	−17%	72,668	−15,832	−18%	8,382,430	−1,668,251	−17%
2021	725,866	162,332	29%	95,501	22,833	31%	10,691,629	2,309,199	28%

**Table 6:** Renal cell and urothelial kidney cancer hospitalisations.

Renal Cell and Urothelial Kidney Cancer	Total Discharges	Annual Change	Chemotherapy (Patients)	Annual Change	Surgical Hospitalisations	Annual Change	Radiotherapy	Annual Change
2015	38,892		1309		5701		959	
2016	39,709	2.10%	1355	3.51%	5804	1.81%	963	0.42%
2017	40,455	1.88%	1331	−1.77%	5790	−0.24%	984	2.18%
2018	41,527	2.65%	1314	−1.28%	6406	10.64%	974	−1.02%
2019	43,080	3.74%	1262	−3.96%	6729	5.04%	991	1.75%
2020	40,206	−6.67%	1164	−7.77%	5956	−11.49%	956	−3.53%
R^2^	0.9754		0.3879		0.8500		0.7655	
Adjusted R^2^	0.9692		0.2348		0.8124		0.7067	
Prognosis for 2020	43,790		1273		6883		996	
Difference Real data vs. prognosis	−3584	−8.18%	−109	−8.56%	−927	−13.47%	−40	−4.02%

**Table 7:** Bladder cancer hospitalisations.

Bladder Cancer	Total Discharges	Annual Change	Chemotherapy (Patients)	Annual Change	Surgical Hospitalisations	Annual Change	Radiotherapy	Annual Change
2015	51,774		2645		20,883		1078	
2016	53,877	4.06%	2754	4.12%	21,831	4.54%	1200	11.32%
2017	55,789	3.55%	2831	2.80%	22,780	4.35%	1215	1.25%
2018	57,498	3.06%	2862	1.10%	23,747	4.24%	1265	4.12%
2019	60,372	5.00%	2990	4.47%	24,903	4.87%	1287	1.74%
2020	57,259	−5.16%	2666	−10.84%	22,649	−9.05%	1212	−5.83%
R^2^	0.992235		0.969243		0.998255		0.880402	
Adjusted R^2^	0.9902		0.9615		0.9978		0.8505	
Prognosis for 2020	62,107		3055		25,815		1353	
Difference Real data vs. prognosis	−4848	−7.81%	−389	−12.73%	−3166	−12.26%	−141	−10.42%

## Data Availability

Data supporting reported results can be found at: https://statystyki.nfz.gov.pl/; https://ezdrowie.gov.pl (accessed on 27 September 2022).
